# Supplementary material for: Are carbon emissions trading and green financial instruments synergistic? -Comprehensive quantitative research based on content analysis
Source: PLoS One. 2024 Mar 7;19(3):e0298601. doi: 10.1371/journal.pone.0298601 (PMC10919601; doi:10.1371/journal.pone.0298601)
Supplement: S1 Data — (ZIP) [file pone.0298601.s001.zip › Data/地级市Z_F工作报告.zip!/║╙─╧/╨φ▓2╩╨/└┤╫╘╣1⁄2╓┌║┼í╛┬φ┐╦╩2╛▌═°í┐_2011.docx]

各位代表：

现在，我代表市人民政府，向大会作工作报告，请予审议，并请各位政协委员提出意见。

一、2010年及“十一五”工作回顾

2010年，在省委、省政府和市委的正确领导下，全市上下深入贯彻落实科学发展观，积极应对复杂经济形势，砥砺奋进，开拓创新，较好地完成了市五届人大五次会议确定的各项任务，经济社会发展继续保持了好的趋势、态势和气势，为“十二五”发展奠定了坚实基础。

（一）经济持续较快增长。预计全市生产总值突破1300亿元，增长13%。地方财政一般预算收入57.4亿元，增长22%。工业增加值850亿元，增长16%。社会消费品零售总额349亿元，增长19.5%。

（二）项目建设进展顺利。“3618”投资促进计划顺利实施，许继风电整机、许都大剧院、魏武大道等一大批重大产业项目、社会事业项目、基础设施项目投产投用，全市100个重点项目完成投资280亿元，带动全社会固定资产投资820亿元。

（三）城镇化建设全面提速。全市城镇化率达到41%。市区重点地段实现了控制性详细规划全覆盖，县（市）村镇体系规划全部通过省级评审。81个城建重点项目和89个城中村改造项目完成投资144.9亿元，拆迁513万平方米，建设安置房400万平方米。高标准编制了《许昌新区空间战略发展规划》，新区173个项目完成投资159亿元。数字化城管系统正式运行，市区基本实现全覆盖、全时段管理。

（四）结构调整步伐加快。二、三产业增加值占生产总值的比重达89.1%。10个产业集聚区实现主营业务收入1000亿元，利税突破130亿元，固定资产投资累计超过300亿元。落实促进产业升级等税收优惠政策，为408户企业抵扣固定资产进项税2亿元，办理各项减免退税等税式财政支出16.2亿元。三大主导产业完成增加值370亿元，占规模工业的53%。科技进步对经济增长的贡献率达54%。全市化学需氧量、二氧化硫排放量分别控制在1.29万吨、3.4万吨以内，市区环境空气质量优良天数完成省定目标。

（五）“三农”工作扎实有效。粮食总产275万吨，连续7年创历史新高。现代农业加快发展，全市花木、蔬菜、中药材种植面积进一步扩大，产业化程度明显提高。畜牧业总产值120亿元，占农业总产值的46.5%。新增市级以上农业产业化龙头企业50家，累计达195家。

（六）招商引资成效显著。“5266”招商引资行动计划扎实推进，签约项目526个，合同引进市外资金889亿元，许昌名嘉广场、温州电气产业园等一批重大产业转型升级项目成功入驻。新批准设立外商投资企业17家，实际利用外资2.5亿美元，增长33%。森源电气、远东传动轴成功上市，利用资本市场融资60亿元。市经济技术开发区被批准为国家级经济技术开发区。荣获全省“对外开放优秀市”。

（七）民生改善富有实效。“十项民生工程”全面完成。全市涉及民生的财政支出达66亿元，占一般预算支出的56.4%，增长19.4%。城镇居民人均可支配收入15010元，农民人均纯收入6806元，分别增长10.2%、8%。全市各金融机构人民币存款余额814.5亿元，贷款余额562.3亿元，分别增长27%、17.3%。

2010年各项目标任务的圆满完成，为辉煌“十一五”划上了圆满的句号，实现了精彩收官。可以说，过去的五年，是许昌经济社会发展总体持续、总体提升、总体协调、总体有效的五年，是许昌历史上经济发展最快、城乡面貌变化最大、人民群众得到实惠最多的五年，也是我们奋发有为、经受考验、夯实基础、团结奋进的五年。

这五年，综合经济实力显著提升。面对复杂多变的经济形势和严峻挑战，大力实施投资促进计划，每年抓好100个投资亿元以上项目；认真开展企业服务年活动，累计为企业解决问题1514个；组织实施招商引资行动计划，举办、参与了一系列重大招商活动，取得并巩固和扩大了战危机、保发展成果，全市经济连续5年保持了较快增长。生产总值提前2年实现突破千亿元的目标，人均生产总值是“十五”末的1.8倍，财政一般预算收入是“十五”末的2.9倍，累计完成全社会固定资产投资是“十五”时期的4.2倍。

这五年，经济发展方式加快转变。大力发展集群经济，在全省率先规划建设产业集聚区，形成了“一带十区二十个产业集群”的发展格局，谋划建设的中原电气谷上升为省级战略。着力构建现代产业体系，制定实施了电力装备、食品、发制品等产业调整振兴规划，重点培育了风电装备、智能电网、电动汽车等五大战略性新兴产业。加快推动农业产业化经营，荣获“全国农业产业化先进市”。积极推进科技创新，连续8年荣获“全国科技进步先进市”。大力发展现代物流、文化旅游、金融保险等现代服务业，荣获“中国金融生态城市”，成功引进交通银行、光大银行、浦发银行。加快企业上市步伐，新增上市企业4家，上市公司数量达到7家，居全省第2位。

这五年，城乡面貌发生巨大变化。全面加快新型城镇体系建设，市区建成区面积达到80平方公里，城镇化率年均增长近2个百分点。启动建设许昌新区并上升为省级战略，为未来发展赢得了新的空间。着力打造区域交通枢纽，高速公路路网密度居全省第2位。扎实开展“四城同创”，着力改善城市形象，荣获“国家森林城市”、“国家卫生城市”、“中国花木之都”、“全国绿化模范城市”。强化城市“三化”管理，成为全省唯一的“数字化城市管理示范城市”。以“饮干净水、走平坦路、建沼气池、用卫生厕”为重点，加快农村基础设施建设，累计解决农村安全饮水80.2万人，新改建农村公路3273公里，建设户用沼气25万口，完成农村改厕9.6万户。

这五年，改革开放取得丰硕成果。扎实推进各项改革，累计完成企事业单位改革改制257家，第一批63家小煤矿已全部签订兼并重组协议，政府机构改革全面完成，教育、卫生、文化等关键领域、关键环节的改革取得重大进展。积极扩大对外开放，成功引进中国平安、国家电网等一批战略投资者，新批准外商投资企业81家，累计出口35.8亿美元，实际利用外资6.7亿美元，分别是“十五”的3倍和4.5倍。创新发展机制，创优发展环境，探索形成了规划例会、项目代建、财政投资评审、重点项目联动和资源配置等一系列科学有效的工作机制，行政审批事项由2035项精简至195项，成为全省行政审批事项最少、收费标准最低的省辖市之一。

这五年，人民群众得到更多实惠。着力打造公共财政，持续加大民生投入。五年来，财政用于民生的支出达到212亿元，占一般预算支出的52.5%。深入实施“创业富民、就业惠民”工程，城镇新增就业39.2万人，新增农村劳动力转移就业139.2万人，动态消除了零就业家庭，被确定为首批“创建国家级创业型城市”。 61.5万名城乡中小学生享受到免费义务教育，2.4万名进城务工人员子女与城市孩子一样，享有接受义务教育的机会。建成标准化村卫生室1486所，新农合参合率达96.3%；城市低保标准提高到每人每月190元以上，农村低保标准提高到每人每年1100元以上；新建、改建农村敬老院228所，五保集中供养率达到60.8%，居全省前列。建设廉租住房24.8万平方米、经济适用房106万平方米、公共租赁住房6.2万平方米。城镇居民人均可支配收入、农民人均纯收入分别是“十五”末的1.9倍和1.8倍。

这五年，民主法治建设加快推进。坚持依法行政，自觉接受人大及其常委会的监督，支持政协参政议政，认真办复人大代表议案建议和政协委员提案1489件。深入开展学习实践科学发展观活动，扎实推进“五个坚持、两转两提”，大力倡导清新简约、务本责实的良好政风。圆满完成“五五”普法任务，行政监察和审计监督更加有力，财政投资评审累计审减资金9.8亿元。政务公开不断扩大，政府网站累计公开信息28400条。社会治安防控网络日益完善，矛盾纠纷调处机制逐步健全，安全生产监管不断加强，连续3年荣获“全省平安建设先进省辖市”。军政军民更加团结，连续6届荣获“全省双拥模范城市”。连续10年被评为全省人口和计划生育工作优秀单位。民族、宗教、外事、侨务、对台工作全面加强，地方史志、档案、统计、体育、地震、气象等工作都取得了新的成绩。

各位代表，回顾五年来的工作，历程极不平凡，成绩来之不易！弥足珍贵的经验尤为需要我们认真总结和坚持：一是只有坚持以科学发展观为统领，牢牢把握发展第一要务，在发展中促转变，在转变中谋发展，才能实现经济社会又好又快发展；二是只有坚持深化改革开放，通过市场运作破解瓶颈制约，通过机制创新推动管理创新，才能为科学发展提供制度保障和动力源泉；三是只有坚持以人为本，让人民群众成为改革发展的受益者，才能打牢社会和谐稳定的基础；四是只有坚持优化政务环境，增强公信力、执行力和约束力，才能不断提升吸引力、竞争力和生产力。

“十一五”时期取得的辉煌成绩，书写了我市全面建设小康社会的崭新一页，标志着许昌站在了历史发展的新起点，步入了蓄势崛起的新阶段。这些成绩的取得，是省委、省政府和市委正确领导的结果，是市人大、市政协大力支持和监督的结果，更是全市人民群策群力、艰苦奋斗的结果。在此，我代表市人民政府，向全市广大干部群众、驻许部队、武警官兵、公安民警表示崇高的敬意！向所有关心、支持许昌现代化建设的各界人士表示衷心的感谢！

在充分肯定成绩的同时，我们也要清醒地看到，我市经济社会发展中仍然存在不少矛盾和问题：产业结构性矛盾突出，服务业发展滞后，全市服务业增加值仅占生产总值的20%；城镇化发展水平不高，全市城镇化率低于全国平均水平7个百分点，居全省靠后位次；重大支撑性项目较少，特别是纳入中央、省计划盘子，能够形成产业优势、促进转型升级的重大项目偏少；人均发展水平和人均公共服务水平仍然偏低，社会矛盾增多，安全生产形势依然严峻，改善民生、维护稳定任务艰巨；政府自身建设有待加强，一些部门职能转变还不到位，一些干部市场运作、推动科学发展的能力有待进一步提高。对此，我们要高度重视，认真加以解决。

二、“十二五”发展的总体思路和目标要求

“十二五”时期是我市全面建设小康社会的关键时期，是深化改革开放、加快转变经济发展方式的攻坚时期。我们必须清醒判断经济社会发展面临的形势，牢牢把握可以大有作为的重大机遇。从外部环境看，经济发展长期向好的趋势没有改变，沿海发达地区生产要素流动和大规模产业转移进一步加快，有利于我们发挥比较优势，大力吸引外部资金、项目、技术和人才，扩大对外开放，拓宽发展空间。从宏观政策看，国家进一步扩大内需、促进中部地区崛起、加大“三农”投入、启动实施“十二五”规划，特别是国家将我市电力装备制造基地建设纳入产业布局，省委、省政府实施中原经济区建设战略，把我市确定为核心区城市，将为我市发展带来更多更大的支持。从自身发展看，我市人均生产总值已经突破4000美元，正处于工业化、城镇化加速发展阶段，产业升级和消费升级步伐加快，内需动力将得到有效释放，有利于经济长期平稳较快发展。特别是我市各级干部驾驭市场经济能力不断增强，市场运作水平明显提高，成为经济社会持续健康发展的不竭动力。我们必须增强加快发展的紧迫感和责任感，抓住用好难得机遇，努力克服不利因素，加快转变经济发展方式，力争各项工作提升进位、走在前列，推动全市经济社会科学发展、转型发展、跨越发展。

“十二五”全市经济社会发展的指导思想是：深入贯彻落实科学发展观，坚持“四个重在”实践要领，以科学发展为主题，以加快转变经济发展方式为主线，以推进新型城镇化为战略重点，坚持走以新型城镇化引领“三化”协调发展的路子，着力改革开放，着力创新创业，着力改善民生，力争实现经济发展速度、城乡居民收入增速高于全省平均水平，发展质量、生态环境好于全省平均水平，在中原经济区建设中奋力开创许昌科学发展、富民强市的新局面。

　“十二五”发展的总体要求可概括为：“三化”协调、“两高两好”、科学发展、富民强市。其中“三化”协调是路径，“两高两好”是目标，科学发展是主题，富民强市是方向，四者共同统一于科学发展的实践，是引领未来5年许昌发展的总纲。

“十二五”全市经济社会发展的主要目标是：生产总值年均增长11%，全社会固定资产投资年均增长16%，地方财政一般预算收入年均增长12%，二、三产业增加值占生产总值的比重达到90%，城镇居民人均可支配收入、农民人均纯收入年均增长10%，外贸进出口总额年均增长20%，外贸出口总额年均增长21%，实际利用外商投资年均增长21%，城镇化率突破50%，单位生产总值能耗完成省定目标任务。在中原经济区中的综合实力和竞争力显著提高，经济发展方式加快转变，人民物质文化生活显著改善，全面建成小康社会、实现富民强市的基础更加牢固。

实现上述目标，我们将重点抓好8项战略任务：一是保持经济平稳较快发展。二是加快经济发展方式转变。三是加快推进城镇化进程。四是加快发展现代农业。五是加快构建自主创新体系。六是积极推动社会事业发展。七是进一步深化改革开放。八是着力维护社会和谐稳定。

《许昌市国民经济和社会发展第十二个五年规划纲要（草案）》及说明已印发大会，请各位代表一并审议。

三、2011年的工作目标和主要任务

2011年是中国共产党成立90周年，也是全面实施“十二五”规划开局之年，做好今年工作意义重大。

今年政府工作的总体思路是：深入贯彻落实科学发展观，认真落实中央、省委和市委经济工作会议精神，按照“开好局、有作为、求突破”的总体要求，坚持“四个重在”实践要领，以科学发展为主题，以加快转变经济发展方式为主线，以推进新型城镇化为战略重点，以项目建设和招商引资为主要抓手，坚持走以新型城镇化引领“三化”协调发展的路子，着力扩大需求，着力调整结构，着力改革开放，着力改善民生，保持经济平稳较快发展，促进社会和谐稳定。

今年经济社会发展的主要预期目标是：生产总值增速高于全省平均水平1个百分点，地方财政一般预算收入增速高于生产总值增速1个百分点，城镇化率增长2个百分点，全社会固定资产投资增长20%，社会消费品零售总额增长16%，外贸出口增长21%，实际利用外商直接投资增长22%，居民消费价格涨幅控制在4%左右，人口自然增长率控制在6.3‰以内，新增城镇就业6万人，城乡居民收入较快增长，节能减排完成省定目标。

实现上述目标，做好今年经济工作，在具体工作方法上：一是更加注重市场运作。把市场运作作为破解难题的根本手段，同时，积极争取上级政策支持，充分发挥财政杠杆作用，采取综合措施，切实解决好经济社会发展的瓶颈约束。二是更加注重项目带动。把项目建设作为各项工作的总抓手，以项目集聚生产要素，以项目促进投资增长，以项目推动结构调整，以项目提升管理水平，增强经济综合竞争力和发展后劲。三是更加注重招商引资。把招商引资作为“一举应多变”的重要举措，围绕城市建设、现代农业和高端产业，强化激励机制，注重招商引资实效，着力解决“钱从哪里来”的问题。四是更加注重优化环境。把优化政务环境作为增创发展优势的重要内容，完善优惠政策，提供优质服务，着力化解矛盾，注重解决难题，不断提升区域核心竞争力。

2011年，重点抓好以下6个方面的工作。

（一）着力扩大需求，保持经济平稳较快增长

继续保持投资适度快速增长，努力扩大消费需求，促进外贸出口稳定增长，协调拉动经济持续快速发展。

持续扩大投资需求。突出抓好项目建设，坚持扩大投资规模与优化投资结构并举。大力实施“3611”投资促进计划，围绕基础产业、基础设施、基础要素3个方面，在工业、现代服务业、社会事业、城市基础设施和城中村改造、农林水利、交通等6个领域，安排100个重点项目，带动全社会固定资产投资1000亿元。工业方面，大力实施中原电气谷风电齿轮箱、温州电气产业园、西继电梯二期、首山焦化30万吨焦油深加工、森源年产30万辆微型电动车、许昌铁矿等60个重点项目，完成年度投资110亿元。现代服务业方面，加快推进许昌航空港产业园、许昌名嘉广场、许昌深国投商业中心、花都温泉度假区、东城区汽车公园等16个重点项目，完成年度投资23亿元。城市建设方面，扎实推进新兴路提升改造、七一路文峰路立交等城建重点项目，以及北关社区、冯庄街片区等城中村改造项目，完成年度投资140亿元。社会事业方面，突出抓好市文博馆、市直公用事业综合服务中心、许昌卫校扩建、河南农大许昌校区、广电大厦等11个重点项目，完成年度投资15亿元。交通电力方面，实施好石武客专许昌段、郑州机场至许昌城际铁路、许禹铁路改造一期、城乡电网建设等6个项目，完成年度投资12亿元。农林水利方面，着力抓好南水北调总干渠许昌段、高产良田、花木基地建设等5个重点项目，完成年度投资20亿元。认真落实市级领导联系重点项目、重点项目联动和资源配置、联审联批、环境监察、进度督查等推进机制，保障项目建设顺利进行。

增强消费拉动能力。认真落实国家、省刺激消费的各项政策措施，继续实施好家电下乡和以旧换新政策，全年力争销售家电下乡产品23万台。开展节假促销活动，繁荣假日经济。提升家政、养老、商业3大社区服务，加快咨询、餐饮、购物配送、农贸市场等便民服务网点建设，培育新的消费热点。大力开拓农村市场，继续实施“万村千乡市场工程”和“新网工程”，新建农村标准超市100个、配送中心1—2个，改建乡镇旗舰店20个以上。规范房地产业健康发展，促进住房合理消费。整顿和规范市场秩序，加强市场价格监管，营造良好的消费环境。

转变外贸增长方式。加大对出口企业的信贷资金、出口退税等支持力度，确保出口稳定增长。积极开拓国际市场，提高新兴市场占有率。优化出口产品结构，巩固发制品出口份额，扩大机电产品、高新技术产品、农副产品出口规模，确保出口总量稳居全省前列。建立健全出口品牌培育、发展和保护机制，增强出口品牌国际竞争力。加强发制品、电子及汽车零配件、超硬材料、轻纺产品等出口基地建设，提高集约化、标准化水平。

加强经济运行调节。准确把握国家宏观政策走向和市场变化，加强经济运行监测分析，科学应对新情况、新问题。坚持市级领导联系企业、企业服务直通车等制度，健全完善企业服务长效机制，市县两级分别抓好100家重点企业，实行年度动态管理。支持企业创业创新，做好重点行业和企业扭亏增盈工作。优化资源配置，全力保障煤电油气运等要素供应。深化政银企合作，强化中小企业金融服务，全年新增银行贷款超额完成省定目标任务。严控涉企评比检查，切实减轻企业负担。

（二）优化产业结构，加快转变经济发展方式

把加快产业转型升级作为推进新型工业化的重要内容，以产业结构优化升级促进经济发展方式转变。

加快产业集聚区发展。深入实施产业集聚区“两年提升推进计划”， 着力建设效益产业集聚区，争创省级创新型产业集聚区，力争实现主营业务收入1300亿元，完成投资300亿元，主营业务收入超百亿元的产业集聚区达到7个。强化产业支撑，围绕主导特色产业，实现招商引资50亿元，在建亿元以上项目达到80个以上。完善基础设施，加快产业集聚区功能性基础设施、生产性和生活性服务设施建设，增强承载能力。全面推进标准化厂房建设，集约节约利用土地，各产业集聚区标准化厂房面积达到30万平方米以上。规范壮大投融资平台，促进投融资平台股权结构多元化发展，平均融资规模达到3亿元以上。继续坚持月通报、季考核、年奖惩的绩效考评机制，落实市县两级产业集聚区联席会议制度，不断完善推进机制，增强制度保障。

促进工业结构优化升级。坚持以高端产业为引领，着力构建现代工业体系。深入实施电力装备、食品、发制品等产业调整振兴规划，提升三大主导、四大特色产业发展水平。加快培育战略性新兴产业，扎实推进风电装备制造、智能电网、电动汽车及关键零部件、超硬材料、生物医药等五大工程。切实增强自主创新能力，突出抓好省重大科技专项和企业研发平台建设，新增3—5个省级企业研发中心，新培育4—5家国家高新技术企业，科技进步对经济增长的贡献率达到55%。扎实做好质量兴市工作，新培育名优产品20个以上。认真落实支持企业发展的政策措施，继续抓好大企业集团、小巨人企业培育工作，推动企业做大做强，力争3家企业销售收入突破100亿元。

积极发展现代服务业。把推动服务业大发展作为产业结构升级的战略重点，努力提高服务业在国民经济中的比重。深入实施“3615”现代物流业提速工程，推进六大物流园区和十大特色产业物流中心建设，现代物流业增加值增长14%。整合文化旅游资源，推进市旅游服务中心、花博园、神垕古镇综合开发等重点项目建设，办好节会活动，全市接待旅游人数和旅游总收入分别增长15%和20%。大力发展金融服务业，组建2家农村商业银行和2—3家村镇银行，推进许昌银行增资扩股，力争引进1—2家外埠股份制商业银行。积极发展信息、中介、商贸、设计等现代服务业。

大力推动企业上市。以培育和推动企业上市为主要抓手，促进企业调整结构，规范管理，上档升级，提升新型工业化水平。加快企业上市步伐，争取1—2家企业成功上市，2家以上企业上报首次公开募股申请，推动2—3家企业进入辅导报备，上市预备队企业保持在30家左右，利用资本市场融资55亿元。进一步简化程序，提高效率，加强指导，为企业上市提供优质服务。

切实抓好节能减排。严格落实节能减排目标责任制、“一票否决制”，完成省定节能减排目标任务。抓好重点行业、重点地区节能降耗。控制污染排放，推进污水处理、垃圾处理和电厂脱硫等项目建设，强力推进清潩河流域综合整治。加强饮用水源地保护，保障人民群众饮水安全。严格环境准入，市级统筹排污总量指标，优先保障重点项目。发展循环经济，争取襄城县煤焦化产业园成为首个省级循环经济标准化示范区。倡导低碳生活，积极开展公共领域节能与新能源汽车示范推广试点工作。加快低碳示范基地建设，争创国家级可再生能源建筑应用示范城市，积极申报国家级低碳试点城市。

（三）统筹城乡发展，全面加快新型城镇化建设

以新型城镇化引领“三化”协调发展，全面加强城镇规划、建设和管理，扎实有效地推进新型城镇化。

提升城乡规划水平。坚持“五规合一、五规同向、五规同步”，构建科学合理、层次清晰的城乡规划体系。修编城市总体规划，提升县（市）城区总体规划，加强分区规划、专项规划和城市设计，完成城市组团通道规划。加大村镇规划编制力度，提升乡镇总体规划，加快编制村庄规划，逐步实现市域规划全覆盖。完善控制性详细规划和修建性详细规划，高标准做好城建重点项目及城中村、棚户区改造规划。建立健全规划技术委员会、规划建设委员会两级审批制度。形成政府组织、公众参与、专家评审、科学决策的长效机制，确保规划的科学性、前瞻性。加大规划执法力度，严格查处违规建设行为。

完善现代城镇体系。坚持“带状城市、组团布局、向心发展、城乡统筹”，着力打造“一中心、五组团”，加快构建中心市区、县市城区、中心镇区、新型农村社区4个层次布局合理、城乡统筹、互促共进的现代城镇体系。切实提升中心城区首位度，着力改造提升老城区、完善提质东城区、增效提速开发区，增强城市综合竞争力。整体推进5个县（市）组团发展，开工建设107国道长葛至新郑段、311国道许昌至襄城段改扩建工程，打造半小时通勤圈、经济圈、生活圈。积极推动中心镇建设，支持基础好、产业优的中心镇率先发展成为小城市。扎实推进新型农村社区建设，尊重群众意愿，充分考虑农民的当前利益和长远生计，率先推进产业集聚区、城市新区和中心城市近郊区村庄改造。

加快许昌新区建设。按照“四区一基地”的功能定位和“主副两中心、四区加两翼”的空间布局，高水平编制许昌新区总体规划，完善专项规划和控制性详细规划，做好主、副中心的城市设计，基本形成许昌新区规划体系。全面启动主中心建设，完善副中心配套功能，推动中原电气谷大发展、出形象。强化项目支撑，全力抓好112个重点项目，完成年度投资110亿元。大力实施北外环东延、魏文路北延、创业大道东延等道路工程，着力推进新区热电厂、供水加压站、医院、学校等基础设施和公共服务设施建设，加快“三村”改造、新型社区和林业生态建设，不断提升承载能力。加快社会事业发展，深化户籍制度改革，完善社会保障制度，实现医疗保险、养老保险全覆盖，把符合条件的农村低保对象全部纳入城镇低保范围。

强化城市建设管理。实施好总投资305亿元的93个城市基础设施项目和51个城中村改造项目，重点抓好积水点改造、污水管网、农贸市场、垃圾分类处理等涉及民生的项目。加大旧城改造力度，力争3年内完成建成区和产业集聚区内城中村改造任务。严格执行土地收储、出让程序，坚持阳光运作，防止“跑冒滴漏”。树立“二级组团”、“区域组团”规划理念，统筹功能配套和公共设施建设。加快交通基础设施建设，推进客运零距离换乘和货运无缝对接，打造综合交通运输体系。以创建全国文明城市为统领，带动各项创建工作全面提升。持续推进城市管理精细化、管理手段数字化、城市夜景灯饰化，深入开展交通秩序、环境卫生、广告设置、私搭乱建等专项整治，进一步提升城市形象。

（四）抓好“三农”工作，促进农业农村经济发展

大兴水利强基础、狠抓生产保供给、力促增收惠民生、着眼统筹添活力，为工业化、城镇化发展提供基础支撑。

切实稳定粮食生产。落实最严格的耕地保护制度，确保基本农田总量不减。不折不扣地落实良种补贴、粮食直补、农机具购置补贴等政策，充分调动农民种粮积极性，力争粮食总产达到275万吨以上。围绕省粮食核心区建设，加快中低产田改造和高产田巩固提升，认真实施25.5万亩高产稳产田项目。深入实施高产创建，突出抓好长葛、鄢陵整体推进和其它县（市）20个乡（镇）重点推进工作。

大力发展现代农业。以公司运作、土地流转、基础设施和新农村建设为抓手，切实提高现代农业发展水平。大力调整农业结构，积极推动种植业结构向果蔬花卉园艺调整、大农业结构向畜牧业调整、农村经济结构向农产品加工调整。加快现代农业示范区建设，着力提升优质粮食、花木、蔬菜、烟草等园区建设水平，全年新增花木面积5万亩、无公害蔬菜5万亩、中药材示范基地2万亩、现代烟草农业示范基地10万亩。大力发展现代畜牧业，改造提升400个标准化规模养殖场和40个养殖小区，畜牧业总产值占农业的比重达到47%。扶持发展龙头企业，培育壮大一批发展前景好、科技含量高、辐射带动能力强的农业产业化龙头企业。

着力完善基础设施。抓住强农惠农政策机遇，加大农村基础设施建设投入，持续改善农业农村生产生活条件。新解决农村居民安全饮水20万人，新改建提升县乡公路300公里，改建危桥500延米，新增户用沼气3万口，改厕5320个。吃透政策，积极争取，注重运作，扎实推进农田水利基本建设，加强水库除险加固、河段险工岁修和骨干防洪河道治理，新发展节水灌溉10万亩，发展和改善有效灌溉7万亩。认真做好第十一届中原花木交易博览会筹备和第八届中国花卉博览会申报工作。切实做好南水北调移民安置和中线工程许昌段建设工作。

加快推进新农村建设。坚持以“建新社区、入新农合、住敬老院、领养老金”为重点，分类指导，分步实施，积极稳妥地推进新农村建设。继续抓好100个新农村示范村建设、67个试点村村容村貌整治提升工作。深入开展农村环境综合整治，力争创建18个省级以上生态村和3个省级以上生态乡镇。切实提高林业生态建设质量、档次和水平，扎实推进生态廊道、村镇绿化和农田防护林体系等重点工程建设，新建31个市级林业生态示范村，完成林业生态建设任务17.8万亩。加大扶贫开发力度，解决1.5万人的脱贫问题。

（五）深化改革开放，增强经济社会发展活力

坚定不移地推进改革开放，以改革破难题，以开放增活力，力争在重点领域和关键环节取得新突破。

扎实做好改革改制。以化解历史遗留问题、引进战略合作者为重点，加快推进38家企事业单位改革改制。全面落实国家基本药物制度，改革医药卫生体制。加快推进灞陵桥等文化事业和专业剧团内部机制改革，促进文化产业发展。积极整合教育资源，组建文博教育集团，深化教育体制改革。强化政府管理创新，推进行政审批、财政投资评审和警务体制改革。深化农村改革，大力推进农用地整治，深入开展村庄整治和城乡建设用地增减挂钩，积极探索农村宅基地有偿退出途径。加快农村土地流转，新发展农民专业合作社200家，加快推进农信社改革步伐，完善提升集体林权主体改革。

有效开展招商引资。把招商引资作为“一举应多变”的重要举措，以大招商促进大发展。组织实施“5368”招商引资行动计划，围绕园区、产业、企业、项目和重点区域5个方面，谋划推介300个重点招商项目，突出抓好60个亿元以上重点项目，合同引进市外资金800亿元。紧盯珠三角、长三角等重点区域，瞄准国内外500强、央企和行业龙头企业，创新方式，强化措施，跟踪问效，着力提高合同履约率、项目开工率、资金到位率。健全完善招商引资考核奖励机制，分层次、大力表彰奖励招商引资先进单位和个人，营造更加浓厚的招商引资氛围。

不断提升开放水平。加快科技、教育、文化、卫生、体育等领域开放步伐，吸引更多境内外资金进入高新技术产业、现代服务业、节能环保和现代农业，参与城镇化和农村基础设施建设，形成宽领域对外开放格局。抢抓国内外产业结构调整和区域性转移重大机遇，用好许昌新区和产业集聚区等平台，围绕构建现代产业体系，更大规模、更高层次、更广范围地承接产业转移。健全完善对外开放体系，力争设立出入境检验检疫局获得审批，申报设立海关取得实质性进展。

（六）保障改善民生，全面促进社会和谐稳定

进一步加大资金投入，继续确定实施“十项民生工程”，加强社会建设，提高公共服务能力，让人民群众得到更多实惠。

积极扩大创业就业。坚持以创业促进就业，认真落实优惠政策措施，发放小额担保贷款5亿元，争创国家级创业型城市。深入实施“全民技能提升”工程，推进“百万培养计划”，完成培训20万人。实施更加积极的就业政策，多渠道增加就业岗位，新增农村劳动力转移就业7万人，下岗失业人员再就业1.2万人，就业困难人员再就业8000人，保持城市零就业家庭动态为零。

大力发展社会事业。坚持优先发展教育事业，继续落实“两免一补”政策，推进农村校舍维修改造，加大市区教育资源整合力度，加快编制幼儿园布点规划，促进基础教育均衡化发展。改善办学条件，着力解决个别学校师资匮乏问题，加快推进高中教育。深入实施职教攻坚计划，加快许昌电气职业学院申办工作，推进许昌工商管理学校新校区建设。加快推进东区医院、建安医院和4所县医院建设，完成703所标准化村卫生室、5个社区卫生服务中心、2个乡镇卫生院建设任务。切实做好重大传染病防治，提高突发公共卫生事件应急处置能力。继续实施公共文化服务体系建设工程，加快36个乡镇综合文化站和657个农家书屋建设。积极发展有线电视、地面数字电视，新增用户3万户。大力推进体育器材进社区和农民体育健身工程，广泛开展全民健身运动。完善落实计划生育利益导向机制，稳定低生育水平，促进人口长期均衡发展。

着力加强社会保障。加大保障性住房建设力度，建设廉租住房17万平方米、经济适用房37.4万平方米、公共租赁住房1.5万平方米，着力解决困难群众住房问题。推进社会保险扩面征缴，企业养老保险新增参保1.3万人，城镇职工医疗保险新增参保5000人，社保基金征缴增速达到9%以上。扎实做好新型农村养老保险试点工作。加强城乡低保规范化建设，做到动态管理下的应保尽保，城市低保标准提高到每人每月210元，农村低保标准提高到每人每年1200元。全面提升农村敬老院建设管理水平，完成60所敬老院升级达标，五保户集中供养率保持在60%以上。

全力维护社会稳定。深入推进平安许昌建设，着力营造安定和谐的经济社会发展环境。以创建“安全许昌”为载体，切实加强煤炭、交通、食品卫生、消防等领域安全监管，深入推进煤炭企业兼并重组，有效防范、坚决遏制重特大安全事故发生。继续改进信访工作，畅通群众诉求渠道，积极化解社会矛盾。完善社会治安大防控体系，强化综合治理和专项整治，依法严厉打击各类违法犯罪活动，增强群众安全感。

加强国防和民兵预备役工作，争创全国双拥模范城，重视和支持工会、共青团、妇联等人民团体工作。认真做好第七届村委会换届选举工作。切实做好外事、侨务、对台工作。继续做好民族、宗教、地方史志、新闻出版、人事、审计、统计、档案、地震、气象、人防等各项工作。

四、全面加强政府自身建设

坚持“三具两基一抓手”，深入开展“五个坚持、两转两提”，以领导方式转变加快发展方式转变，着力提高推动科学发展的能力和水平。

（一）按章理事，增强依法行政能力。严格按照法定权限和程序行使权力、履行职责。依法接受人大及其常委会的法律监督、工作监督，自觉接受政协的民主监督，认真办理人大代表建议议案和政协委员提案。健全规章制度，简化办事程序，坚持用制度管权、按制度办事、靠制度管人。规范行政执法行为，强化执法监察、效能监察和廉政监察，推进严格执法、公正执法、文明执法。

（二）牢记宗旨，增强为民执政能力。恪守为民之责、善谋富民之策、多办利民之事，切实解决好柴米油盐酱醋茶、衣食住行教医保等民生问题。坚持把群众高兴不高兴、拥护不拥护、赞成不赞成、满意不满意作为评判各项工作的根本标准，让人民群众共享改革发展成果。扩大政务公开范围，建立完善110应急联动系统、12319城市管理服务热线等多种便民服务体系。加强公务员队伍教育和管理，继续开展争做“人民满意的公务员”活动。

（三）转变作风，增强高效施政能力。始终保持强烈的责任感和使命感，做到责随职走、心随责走。坚持求真务实，认真调查研究，遵循客观规律，科学民主决策，力争不走错路、少走弯路。用好用活注重运作的工作方法，坚持各项行之有效的推进机制，说到做到，说好做好。大力整治行政不作为、慢作为、乱作为，确保令行禁止、政令畅通。精简会议文件，减少评比检查，腾出更多的精力抓落实。

（四）自警自律，增强廉洁从政能力。严格执行党风廉政建设责任制和领导干部廉洁从政准则，加强对重点环节和重点部位的制约和监督，使权力运行到哪里，监督就延伸到哪里。加强政府采购管理，全面推行公务卡制度，扩大“小金库”治理范围。继续大力倡导清新简约、务本责实的良好政风，坚决反对铺张浪费。坚决查处违纪违法案件，坚决惩治腐败分子，坚决纠正损害群众利益的不正之风。

各位代表，站在新起点，实现新跨越，是时代赋予我们的光荣使命和神圣职责。让我们在中共许昌市委的正确领导下，深入贯彻落实科学发展观，加快推动经济发展方式转变，紧紧依靠、团结带领全市人民，抢抓机遇，真抓实干，努力把“十二五”规划的宏伟蓝图变为美好现实，为开创许昌更加美好的明天而努力奋斗！
